# Differences in Enzymatic Properties of the *Saccharomyces kudriavzevii* and *Saccharomyces uvarum* Alcohol Acetyltransferases and Their Impact on Aroma-Active Compounds Production

**DOI:** 10.3389/fmicb.2016.00897

**Published:** 2016-06-07

**Authors:** Jiri Stribny, Amparo Querol, Roberto Pérez-Torrado

**Affiliations:** Food Biotechnology Department, Instituto de Agroquímica y Tecnología de Alimentos – Consejo Superior de Investigaciones Científicas, ValenciaSpain

**Keywords:** yeasts, *Saccharomyces*, *ATF1*, *ATF2*, higher alcohols, acetate esters, synthetic wine must

## Abstract

Higher alcohols and acetate esters belong to the most important yeast secondary metabolites that significantly contribute to the overall flavor and aroma profile of fermented products. In *Saccharomyces cerevisiae*, esterification of higher alcohols is catalyzed mainly by the alcohol acetyltransferases encoded by genes *ATF1* and *ATF2*. Previous investigation has shown other *Saccharomyces* species, e.g., *S. kudriavzevii* and *S. uvarum*, to vary in aroma-active higher alcohols and acetate esters formation when compared to *S. cerevisiae*. Here, we aimed to analyze the enzymes encoded by the *ATF1* and *ATF2* genes from *S. kudriavzevii* (*SkATF1*, *SkATF2*) and *S. uvarum* (*SuATF1*, *SuATF2*). The heterologous expression of the individual *ATF1* and *ATF2* genes in a host *S. cerevisiae* resulted in the enhanced production of several higher alcohols and acetate esters. Particularly, an increase of 2-phenylethyl acetate production by the strains that harbored *ATF1* and *ATF2* genes from *S. kudriavzevii* and *S. uvarum* was observed. When grown with individual amino acids as the nitrogen source, the strain that harbored *SkATF1* showed particularly high 2-phenylethyl acetate production and the strains with introduced *SkATF2* or *SuATF2* revealed increased production of isobutyl acetate, isoamyl acetate, and 2-phenylethyl acetate compared to the reference strains with endogenous *ATF* genes. The alcohol acetyltransferase activities of the individual Atf1 and Atf2 enzymes measured in the cell extracts of the *S. cerevisiae atf1 atf2 iah1* triple-null strain were detected for all the measured substrates. This indicated that *S. kudriavzevii* and *S. uvarum* Atf enzymes had broad range substrate specificity as *S. cerevisiae* Atf enzymes. Individual Atf1 enzymes exhibited markedly different kinetic properties since SkAtf1p showed *c.* twofold higher and SuAtf1p *c.* threefold higher *K*_m_ for isoamyl alcohol than ScAtf1p. Together these results indicated that the differences found among the three *Saccharomyces* species during the aroma-active acetate ester formation may be due, to some extent, to the distinct properties of Atf enzymes.

## Introduction

Amino acid metabolism, which occurs during yeast fermentation processes, plays an important role in fragrance, flavor and food industries. Its importance arises from the fact that amino acid catabolism leads to the production of higher alcohols, which are precursors of acetate esters (Dickinson et al., 1997, 1998, 2000, 2003; Hazelwood et al., 2008). Higher alcohols and acetate esters belong to the principal aroma-active substances that affect the complex flavor of fermented products (Welsh et al., 1989; Verstrepen et al., 2003a). The most significant acetate esters include isobutyl acetate (fruity-like aroma), isoamyl acetate (banana), and 2-phenylethyl acetate (flowery, rose-like), which result from the esterification of the corresponding higher alcohols: i.e., isobutanol (derived from valine), isoamyl alcohol (derived from leucine), and 2-phenylethanol (derived from phenylalanine), respectively (Nykanen, 1986; Lambrechts and Pretorius, 2000; Styger et al., 2011). The reaction of higher alcohols with acetyl-CoA, which results in acetyl esters, is catalyzed in *S. cerevisiae* by alcohol acetyltransferases I and II (AATase I and II, EC 2.3.1.84), codified by the *ATF1* and *ATF2* genes, respectively (Yoshioka and Hashimoto, 1981; Malcorps et al., 1991; Minetoki et al., 1993; Fujii et al., 1994; Nagasawa et al., 1998). It has been reported that Atf1p and Atf2p catalyze the acetylation of a wide variety of higher alcohols. Mainly isoamyl alcohol, but also other alcohols, such as propanol, isobutanol, hexanol, and 2-phenylethanol, are esterified by Atf1p and Atf2p (Verstrepen et al., 2003b). Nevertheless, the contributions of each enzyme seem to differ. The deletion and overexpression of *ATF2* resulted only in minor changes in the formation of acetate esters, which indicated that the *ATF2*-encoded enzyme plays only a minor role compared with the *ATF1*-encoded enzyme (Verstrepen et al., 2003b; Lilly et al., 2006).

The most common yeast species employed in industrial applications, including food, flavor, and fragrance production, is *S. cerevisiae*. Our previous studies reported differences in the production of the higher alcohols and acetate esters directly derived from amino acids by *S. kudriavzevii* and *S. uvarum* compared to *S. cerevisiae* (Stribny et al., 2015). The analysis revealed, for instance, that *S. kudriavzevii* produced larger amounts of higher alcohols than *S. cerevisiae*, whereas *S. uvarum* excelled in acetate esters production. Similarly, differences in aroma compounds production by the three *Saccharomyces* species were observed during wine must fermentations (Gamero et al., 2013). To gain a better insight into the molecular aspects of the aforementioned differences, our recent study (Stribny et al., 2016) aimed to explore the nucleotide divergences in 23 ortholog genes (and, consequently, in the corresponding enzymes) from *S. kudriavzevii/S. uvarum* vs. *S. cerevisiae* involved in amino acid catabolism, which lead to aroma-active higher alcohols and esters production. An *in silico* analysis using Grantham scoring, which quantitatively evaluates (dis)similarity in amino acids substitutions on the basis of physiochemical properties (composition, polarity, and molecular volume; Grantham, 1974), detected the *ATF1*- and *ATF2*-encoded proteins among the hits with the highest Grantham scores.

In the present work, we cloned *ATF1* and *ATF2* genes from *S. kudriavzevii* and *S. uvarum* into *S. cerevisiae* to examine and compare their effect on higher alcohols and acetate esters production. Kinetic parameters together with substrate specificities of the encoded enzymes were also analyzed and compared with *S. cerevisiae*.

## Materials and Methods

### Yeast Strains

The yeast strains used in this study are listed in **Table 1**. *S. cerevisiae* Ta, a haploid strain derived from commercial wine strain T73, was previously constructed in the laboratory of A. Querol. Standard complex media (0.5% peptone, 2% glucose, and 0.5% yeast extract) or SC-Ura medium [0.67% YNB, 2% glucose, 1.92 g/L Drop-out –Ura (Formedium, Norfolk, UK)] were used to grow stock cultures. DNA isolation, restriction, and gel electrophoresis were carried out by standard genetic methods. Strains were transformed by the lithium acetate procedure (Gietz and Woods, 2002).

**Table 1:** List of the yeast strains used in this study.

Strain	Species	Description	Reference
T73	*S. cerevisiae*	Wine strain, Alicante, Spain	Querol et al., 1992
IFO1802	*S. kudriavzevii*	Type strain, NCBI	Kaneko and Banno, 1991
CECT12600	*S. uvarum*	Wine strain, Alicante, Spain	Spanish culture collection (CECT)
Ta	*S. cerevisiae*	T73*ho*Δ*::loxP*	A. Querol
JET02	*S. cerevisiae*	Ta *atf1*Δ::*NAT1*	This study
JET02Sk	*S. cerevisiae*	Ta *atf1*Δ::*SkATF1-kX*	This study
JET02Su	*S. cerevisiae*	Ta *atf1*Δ::*SuATF1-kX*	This study
JET02Sc	*S. cerevisiae*	Ta *atf1*Δ::*ScATF1-kX*	This study
JET03	*S. cerevisiae*	Ta *atf2*Δ::*NAT1*	This study
JET03Sk	*S. cerevisiae*	Ta *atf2*Δ::*SkATF2-kX*	This study
JET03Su	*S. cerevisiae*	Ta *atf2*Δ::*SuATF2-kX*	This study
JET03Sc	*S. cerevisiae*	Ta *atf2*Δ::*SuATF2-kX*	This study
BY4741atf1atf2iah1	*S. cerevisiae*	*MAT*a *leu2-*Δ*0 his3-*Δ1 *met15-*Δ*0 ura3-*Δ*0 atf1*Δ*1*::loxP *atf2*Δ*1*::loxP *iah1*Δ*1*::loxP	Uber-Garcia, 2005
CLpSkATF1	*S. cerevisiae*	BY4741atf1atf2iah1 pG-SkATF1-TDH3p	This study
CLpSuATF1	*S. cerevisiae*	BY4741atf1atf2iah1 pG-SuATF1-TDH3p	This study
CLpScATF1	*S. cerevisiae*	BY4741atf1atf2iah1 pG-ScATF1-TDH3p	This study
CLpSkATF2	*S. cerevisiae*	BY4741atf1atf2iah1 pG-SkATF2-TDH3p	This study
CLpSuATF2	*S. cerevisiae*	BY4741atf1atf2iah1 pG-SuATF2-TDH3p	This study
CLpScATF2	*S. cerevisiae*	BY4741atf1atf2iah1 pG-ScATF2-TDH3p	This study

### Plasmid and Strain Construction

The *S. kudriavzevii ATF1* and *ATF2* genes (*SkATF1* and *SkATF2*) were amplified from the genomic DNA of *S. kudriavzevii* IFO1802. The *S. uvarum ATF1* and *ATF2* genes (*SuATF1* and *SuATF2*) were amplified from the genomic DNA of *S. uvarum* CECT12600. The *S. cerevisiae ATF1* and *ATF2* genes (*ScATF1* and *ScATF2*) were amplified by the PCR from the genomic DNA of *S. cerevisiae* T73. The primers that we used are listed in **Table 2**. PCR fragments were then independently cloned into the pGREG526 vector (Jansen et al., 2005), previously cut with NotI/SalI. Before the start codon of the individual *ATF* genes within the pGREG526 vectors, the *TDH3* promoter (amplified by PCR from genomic DNA from the S288C strain) was inserted by homologous recombination. The resulting plasmids are listed in **Table 3**. The constructed plasmids that contained *ATF* genes behind the *TDH3* promoter were then introduced into the BY4741atf1atf2iah1 strain (**Table 1**) used in the enzyme assays.

**Table 2:** The primers used in this study.

Primer	Sequence 5′–3′
**Cloning into pGREG526**	
SkATF1-F	CCTAGTACGGATTAGAAGCCGCCGAGCGGGTGACATCTTCAAAAGCCTCCTCATAC
SkATF1-R	GCGTGACATAACTAATTACATGACTCGAGGTCGACGCCTAAGGAATGACGATACC
SuATF1-F	CCTAGTACGGATTAGAAGCCGCCGAGCGGGTGACAACAAAACCATAACCGAATACG
SuATF1-R	GCGTGACATAACTAATTACATGACTCGAGGTCGACCGGCTAAAAGAACGATACAA
ScATF1-F	CCTAGTACGGATTAGAAGCCGCCGAGCGGGTGACAGGTACTCATCGTAAAAGATTGC
ScATF1-R	GCGTGACATAACTAATTACATGACTCGAGGTCGACTAACCAACCAAAGCCGAG
SkATF2-F	CCTAGTACGGATTAGAAGCCGCCGAGCGGGTGACATTATCACCAGACGGCTCAC
SkATF2-R	GCGTGACATAACTAATTACATGACTCGAGGTCGACGCTCTGTCCGATACACCG
SuATF2-F	CCTAGTACGGATTAGAAGCCGCCGAGCGGGTGACAATCACCAAAGTAACCACCAT
SuATF2-R	GCGTGACATAACTAATTACATGACTCGAGGTCGACATACCGCTTCCTTGCTGT
ScATF2-F	CCTAGTACGGATTAGAAGCCGCCGAGCGGGTGACAAGGAAGCACGTCAGAAAAAG
ScATF2-R	GCGTGACATAACTAATTACATGACTCGAGGTCGACGCTCTGTCCGATACACTGC
***ATF1/ATF2*** **deletion cassettes**	
TaATF1-NAT1-F	ATGAATGAAATCGATGAGAAAAATCAGGCGCCCGTGCAACGGTGTTTAGGTCGATGCCATC
TaATF1-NAT1-R	CTAAGGGCCTAAAAGGAGAGCTTTATAAATGGAGCAAAGCGGATGGCGGCGTTAGTATCG
TaATF2-NAT1-F	ATGGAAGATATAGAAGGATACGAACCACATATCACTCAAGGGTGTTTAGGTCGATGCCATC
TaATF2-NAT1-R	TTAAAGCGACGCAAATTCGCCGATGGTTTGGTAGAAGAGCGGATGGCGGCGTTAGTATCG
**Integration fragments**	
pGSkATF1f	CTTCATCAGTATCACAAATACCATCAATTTATCAGCTCTCATGACTAAAATCAGCGAAGAG
pGSuATF1f	CTTCATCAGTATCACAAATACCATCAATTTATCAGCTCTCATGAATACCTATAGTGAAAA
pGScATF1f	CTTCATCAGTATCACAAATAC
pG-ATF1-R	TCATATTGTCGAATAATATCAGTCAAGCATCATGTGAGATCTCACTATAGGGCGAATTGG
pGSkATF2f	CGAATAATAACTTCAGCAATAAAAATTGTCCAGGTTAATTATGGATGATATAGAGGAATAC
pGSuATF2f	CGAATAATAACTTCAGCAATAAAAATTGTCCAGGTTAATTATGGATAGTTTAGAGGAATAC
pGScATF2f	CGAATAATAACTTCAGCAAT
pG-ATF2-R	TCGGCCGAGCTATACGAAGGCCCGCTACGGCAGTATCGCACTCACTATAGGGCGAATTGG
**Integration of** ***TDH3*** **promoter**	
pG526-TDH3p-Fa	GAGCTGATACCGCTCGCCGCAGCCGAACGACCGAGCGCAGCAGTTCGAGTTTATCATTATC
pG-TDH3p-CF1-R	CTGATAAATTGATGGTATTTGTGATACTGATGAAGCGAAACTAAGTTCTTGGTGTT
pG-TDH3p-KF1-R	GGTAGATTGATGATATTTGTTATACTGATGAGTGGCGAAACTAAGTTCTTGGTGTT
pG-TDH3p-UF1-R	GTTGGATTGATGGTTTTGTTAAACTGCTAAATTGGCGAAACTAAGTTCTTGGTGTT
pG-TDH3p-CF2-R	CCTGGACAATTTTTATTGCTGAAGTTATTATTCGTCGAAACTAAGTTCTTGGTGTT
pG-TDH3p-KF2-R	CGCAATTTTTTGTTCGTTTGAAGATCTAGTAGAGCCGAAACTAAGTTCTTGGTGTT
pG-TDH3p-UF2-R	GCAGTTTTTTGTTCTGAAACCTATTGTTCGTTTGTCGAAACTAAGTTCTTGGTGTT
**Diagnostic**	
T73ATF1-UF	GGTACTCATCGTAAAAGATTGC
T73ATF2-UF	AGGAAGCACGTCAGAAAAAG
K2	GGGACAATTCAACGCGTCTG
K3	CCTCGACATCATCTGCCC
SkATF1-R1	GCCTAAGGAATGACGATACC
SuATF1-R1	CGGCTAAAAGAACGATACAA
ScATF1-R1	TTTGTGATACTGATGAAGTGCCG
SkATF2-R1	GCTCTGTCCGATACACCG
SuATF2-R1	ATACCGCTTCCTTGCTGT
ScATF2-R1	GCTCTGTCCGATACACTGC

**Table 3:** The plasmids used in this study.

Plasmid name	Description	Reference
pGREG526	*URA3*, *kanMX*, *AmpR*	Jansen et al., 2005
**pGREG526 containing** ***ATF*** **orthologs**		
pG-SkATF1-kX	Containing *S. kudriavzevii ATF1*	This study
pG-SuATF1-kX	Containing *S. uvarum ATF1*	This study
pG-ScATF1-kX	Containing *S. cerevisiae ATF1*	This study
pG-SkATF2-kX	Containing *S. kudriavzevii ATF2*	This study
pG-SuATF2-kX	Containing *S. uvarum ATF2*	This study
pG-ScATF2-kX	Containing *S. cerevisiae ATF2*	This study
**pGREG526 harboring** ***ATF*** **orthologs with the** ***TDH3*** **promoter**		
pG-SkATF1-TDH3p	Containing *S. kudriavzevii ATF1*	This study
pG-SuATF1-TDH3p	Containing *S. uvarum ATF1*	This study
pG-ScATF1-TDH3p	Containing *S. cerevisiae ATF1*	This study
pG-SkATF2-TDH3p	Containing *S. kudriavzevii ATF2*	This study
pG-SuATF2-TDH3p	Containing *S. uvarum ATF2*	This study
pG-ScATF2-TDH3p	Containing *S. cerevisiae ATF2*	This study

The strategy of replacing endogenous *ATF1* and *ATF2* in the Ta genome with the corresponding *ATF* genes from *S. kudriavzevii* or *S. uvarum* involved two steps (i) deletion of either *ATF1* or *ATF2* gene; and (ii) integration of the *S. kudriavzevii* or *S. uvarum* orthologs into the locus. The *ATF* genes deletions in the Ta genome were performed by integrating a nourseothricin resistance cassette by homologous recombination. Deletion cassettes were amplified using pAG25 (Goldstein and McCusker, 1999) as a template and specific primers (**Table 2**). The resulting strains were named JET02 (*atf1*Δ) and JET03 (*atf2*Δ). The integration of *SkATF1* into JET02 is described to illustrate the second step. The integration of the other orthologs was performed in the same way. The integration cassette was amplified from plasmid pG-SkATF1-kX with primers pGSkATF1f and pG-ATF1-R. The resulting PCR fragment included the *SkATF1* gene, followed by kanamycin resistance marker, which was used for selection in the subsequent JET02 strain transformation. The final Ta mutant that held *SkATF1* gene was named JET02Sk. The same procedure was carried out with *ScATF1* (and *ScATF2*), which resulted in the restoration of the endogenous *ATF1* (and *ATF2*) gene by the undergone process. This strain, named JET02Sc, was used as a reference in the subsequent assays.

### Cultivation to Study the Production of the Higher Alcohols and Acetate Esters That Derived from the Corresponding Amino Acids

Cultivations were performed in triplicate with a synthetic medium that contained 0.17% YNB w/o AAs and (NH_4_)_2_SO_4_ (BD DIFCO^TM^, Madrid, Spain) and 2% glucose as the carbon source, as previously described (Stribny et al., 2015) with minimal modifications. Media were supplemented by individual amino acids leucine, phenylalanine, and valine as the nitrogen source. Concentrations were proportional to 5 g/L (NH_4_)_2_SO_4_ to obtain the same nitrogen content, as follows: 10 g/L leucine, 12.5 g/L phenylalanine, 8.9 g/L valine (Bolat et al., 2013).

Starter cultures were prepared by pregrowing yeast in 15-mL tubes that contained 4 mL of standard complex media. Before inoculating the experimental culture, the grown precultures were washed with water and resuspended in the same synthetic medium (with a certain nitrogen source), as used in the assay. Cells were resuspended in a volume that allowed an OD_600_ of 1.7 to be achieved. These precultures (100 μL) were used to inoculate 1.6 mL of synthetic media, when the initial OD_600_ was 0.1. Cultivation was performed in 96-well plates with 2-mL-deep wells. Wells were covered by microplate sealer (Greiner bio-one, Frickenhausen, Germany) to allow yeast growth and avoid evaporation and loss of volatile flavor compounds. Cultures were incubated for 5 days at 25°C. The individual 1.7-mL cultures were later transferred to 2-mL tubes and were stored at -20°C for the analysis.

### Yeast Growth Analysis

Yeast cells growth was followed using a 96-well plate. Synthetic media were supplemented with amino acids as described above. Then 100 μL of media were inoculated in a well with 2 μL of cell suspension with OD_600_ = 1. Growth was monitored in a Spectrostar Nano absorbance reader (BMG Labtech, Ortenberg, Germany).

### Synthetic Wine Must Fermentation

A synthetic wine must was prepared as described previously (Riou et al., 1997), but with 200 g/L of reducing sugars (100 g/L glucose + 100 g/L fructose) and with no anaerobic factors (Beltran et al., 2004). Nitrogen source 300 mg N/L was composed of amino acids (180 mg/L) and NH_4_Cl (120 mg/L). The composition of the amino acids mixture was that described by Beltran et al. (2004). The following mineral salts were used: KH_2_PO_4_ 750 mg/L, K_2_SO_4_ 500 mg/L, MgSO_4_ 250 mg/L, CaCl_2_ 155 mg/L, NaCl 200 mg/L, MnSO_4_ 4 mg/L, ZnSO_4_ 4 mg/L, CuSO_4_ 1 mg/L, KI 1 mg/L, CoCl_2_ 0.4 mg/L, H_3_BO_3_ 1 mg/L, (NH_4_)_6_Mo_7_O_24_ 1 mg/L. The following organic acids were used: malic acid 5 g/L, citric acid 0.5 g/L, and tartaric acid 3 g/L. The following vitamins were used: myo-inositol 20 mg/L, calcium pantothenate 1.5 mg/L, nicotinic acid 2 mg/L, chlorohydrate thiamine 0.25 mg/L, chlorohydrate pyridoxine 0.25 mg/L, and biotin 0.003 mg/L. The final pH was adjusted to 3.3 with NaOH.

Fermentations were performed in 250-mL glass bottles that contained 200 mL of synthetic must. Fermentations were done in triplicate at 25°C with continuous orbital shaking (150 rpm). Flasks were closed with Müller valves and monitored by weight loss until a constant weight was obtained. Immediately after fermentation ended, yeast cells were removed by centrifugation, and the contents of higher alcohols and esters in the supernatants were analyzed by gas chromatography.

### Higher Alcohols and Esters Determination

The samples stored in the 2-mL tubes were centrifuged (13,000 rpm, 2 min) and 1.5 mL of the supernatant was transferred to the 15-mL vials with 0.35 g of NaCl. The 20-μl volume of 2-heptanone (0.005%) was added as an internal standard. Higher alcohols and esters were analyzed by the headspace solid phase microextraction (HS-SPME) technique using a 100-μm poly-dimethylsiloxane (PDMS) fiber (Supelco, Sigma–Aldrich, Madrid, Spain). Solutions were maintained for 2 h at 25°C to establish the headspace-liquid equilibrium. The fiber was inserted through a vial septum into the headspace and was held for 7 min. The fiber was then inserted into the gas chromatograph inlet port for 4 min at 220°C with helium flow (1 mL/min) to desorb analytes. A Thermo Science TRACE GC Ultra gas chromatograph with a flame ionization detector (FID) was used, equipped with an HP INNOWax 30 m × 0.25 m capillary column coated with a 0.25-m layer of cross-linked polyethylene glycol (Agilent Technologies, Valencia, Spain). The oven temperature program was: 5 min at 35°C, 2°C/min to 150°C, 20°C/min to 250°C and 2 min at 250°C. The detector temperature was kept constant at 300°C. A chromatograph was recorded by the ChromQuest program. Volatile compounds were identified by the retention time for reference compounds. Quantification of the volatile compounds was determined using the calibration graphs of the corresponding standard volatile compounds.

### Preparation of Yeast Cell Extracts

Cell extracts were prepared as described previously (Rojas et al., 2002) with minor modifications. Yeasts were grown in SC-Ura at 25°C up to the late log phase and by taking optical density at 600 nm as a reference. Yeast cells were collected by centrifugation, washed twice with cold 0.85% NaCl solution and resuspended in ice-chilled disruption buffer [10 mM potassium phosphate, pH 7.5, which contained 0.8 mM MgCl_2_, 10% (w/v) glycerol, 5 mM DTT]. Protease inhibitor cocktail tablet Complete Mini, EDTA-free (Roche Diagnostics, Mannheim, Germany) was also added to the disruption buffer (one tablet per 10 mL). Disruption was performed with glass beads in a MillMix 20 Bead Beater cell disrupter (Domel Tehtnica, Zelezniki, Slovenia) at a frequency of 30 s^-1^ in 1-min intervals over a 10-min period. The resulting homogenate was centrifuged at 15,000 *g* for 30 min at 4°C and the resulting supernatant was used to assay alcohol acetyltransferase activity.

The protein concentrations in the cell extracts were estimated by the Bradford method (Bradford, 1976) using bovine serum albumin as a standard.

### Enzyme Assays

Enzyme assays were performed in a 25-ml glass syringe provided with a Luer lock cap, as described previously (Rojas et al., 2002) with minor modifications. Reaction mixtures consisted in glycerol buffer [50 mM potassium phosphate, pH 7.5, 10% (w/v) glycerol] that contained higher alcohol as a cosubstrate, glycerol buffer that contained acetyl-CoA as the other cosubstrate and a cell extract. The higher alcohol, acetyl-CoA and cell extract showed the following volume ratio 1:0.1:0.4. The final volume was determined by the number of samples (1.5 mL). Isoamyl alcohol (at a final concentration of 0.01–100 mM), isobutanol (60 mM) or 2-phenylethanol (30 mM) was used as the substrate together with acetyl-CoA (0.8 mM). Substrate concentrations were determined according to Rojas (2002). After adding all the components, entrapped air was removed by the plunger and the syringe was attached to an orbital shaker. After 30 min, the 1.5-mL samples were transferred to 15-mL vials with 0.35 g NaCl for ester quantification with gas chromatography. Enzyme activity was stopped by adding 60 μL of a saturated KSCN solution.

### Statistical Analysis

The presented values are averages of biological triplicates with standard errors. The differences between the measured volatile compounds were determined by a one-way ANOVA, followed by Tukey’s HSD test (statistical level of significance was set at *P* ≤ 0.05). The analysis was performed using the STATISTICA 7.0 software (StatSoft, Inc., Tulsa, OK, USA).

## Results

### Impact of the *ATF1* and *ATF2* Homologous Genes on the Formation of Higher Alcohols and Esters

To verify the effect of the Atf1 and Atf2 enzymes from *S. kudriavzevii* and *S. uvarum* on the final content of higher alcohols and/or acetate esters, individual genes (*SkATF1, SuATF1, SkATF2*, and *SuATF2*) were cloned into the locus of *ATF1* or *ATF2* in a haploid strain of the wine *S. cerevisiae* T73 strain, maintaining in all strains the wild *S. cerevisiae* promoter. The constructed mutants were named JET02Sk, JET02Su, JET03Sk, and JET03Su, respectively (**Table 1**). The formation of the major aroma-active higher alcohols and acetate esters was compared to that of *S. cerevisiae*. To exclude any other mutations that may have occurred in the gene replacement step, the original *ATF1* (or *ATF2*) gene was returned to its native position which resulted in the strain JET02Sc (or JET03Sc) that was then used as a reference in the aroma comparison.

**FIGURE 1:**
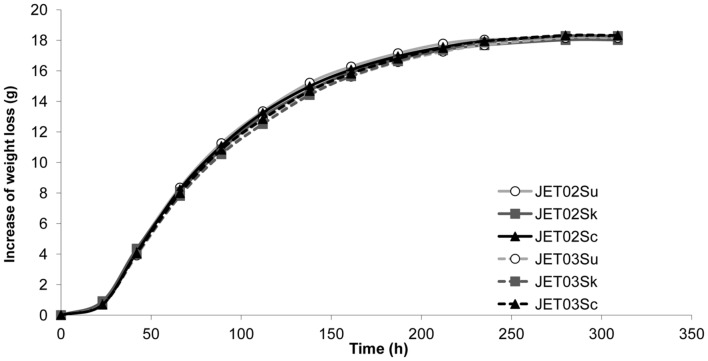
**Progress of synthetic wine must fermentation.** Fermentations were monitored by weight loss until a constant weight was achieved.

**FIGURE 2:**
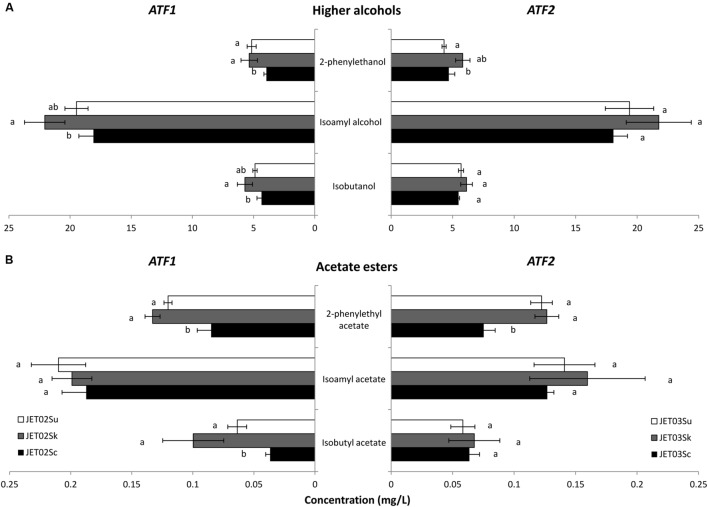
**Production of higher alcohols **(A)** and acetate esters **(B)** by the JET02-derived strains that harbored the individual *ATF1* orthologs (left-oriented bars) and by the JET03-derived strains that harbored the individual *ATF2* orthologs (right-oriented bars) during synthetic wine must fermentation.** The statistically significant differences among the strains were determined independently for each compound and are indicated by labels beside the columns.

Synthetic wine must fermentations by the strains with the different *ATF* genes were performed, and the production of higher alcohols and acetate esters was analyzed. Weight loss monitoring showed all the strains to have similar fermentation rates with no significant differences (**Figure 1**). Regarding aroma compounds production, the most significant differences were observed during 2-phenylethyl acetate production (**Figure 2**). The strains that harbored *ATF1* or *ATF2* from *S. kudriavzevii* and *S. uvarum* produced almost twofold larger amounts of 2-phenylethyl acetate compared to the corresponding reference strains with *ScATF1* or *ScATF2*. Strain JET02Sk also revealed *c.* threefold and JET02Su *c.* twofold larger amounts of isobutyl acetate than JET02Sc. During synthetic must fermentation several ethyl esters (ethyl hexanoate, ethyl decanoate, and ethyl octanoate) were also detected. Nevertheless, the comparison of the concentrations of the ethyl esters produced by the strains with the different *ATF* genes did not reveal any significant differences (data not shown).

**FIGURE 3:**
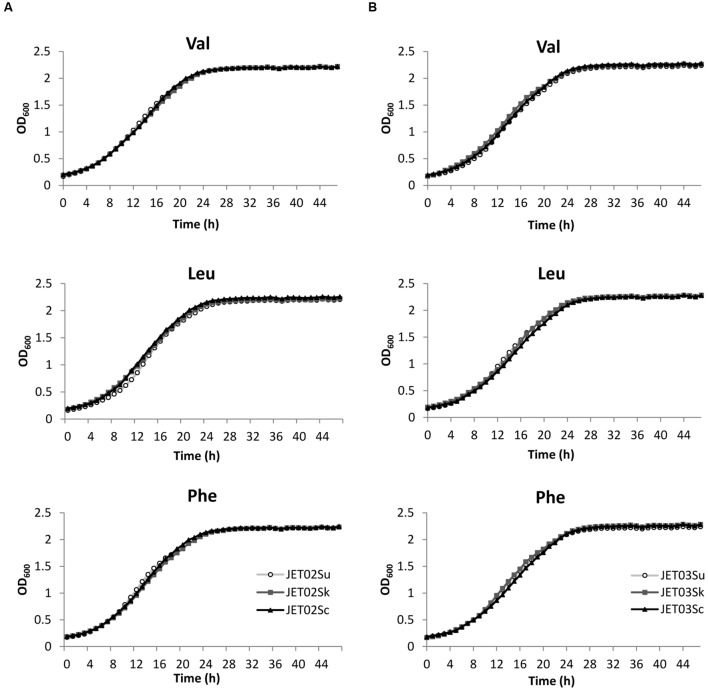
**Growth of the JET02-derived strains that harbored the individual *ATF1* orthologs **(A)** and of the JET03-derived strains that harbored the individual *ATF2* orthologs **(B)** with the indicated amino acids as the nitrogen source**.

**FIGURE 4:**
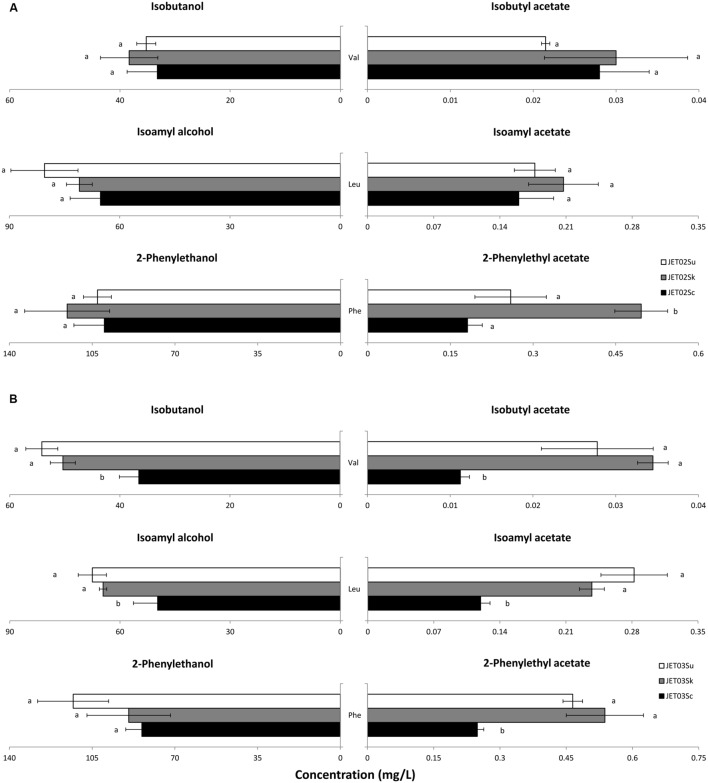
**Production of the higher alcohols and esters derived from the corresponding amino acidic precursors used as the nitrogen source (indicated in the graphs). (A)** Production of the higher alcohols and esters by the JET02-derived strains that harbored the individual *ATF1* orthologs. **(B)** Production of the higher alcohols and esters by the JET03-derived strains that harbored the individual *ATF2* orthologs. The statistically significant differences among the species were determined independently for each nitrogen source and are indicated by labels beside the columns.

Since aroma active higher alcohols, and subsequently acetate esters, derive directly from their amino acidic precursors, our aim was to verify whether, and to what extent, the individual *ATF* genes played a role in the production of higher alcohols and acetate esters from their corresponding amino acidic precursors. Valine, leucine, or phenylalanine was therefore used individually as the sole nitrogen source in the cultivations, and subsequently production of the corresponding higher alcohols and their esters was analyzed. In the assay, all the strains presented a normal growth pattern under these conditions (**Figure 3**). This confirmed their capability to use these individual amino acids as the sole nitrogen source. The analysis of the higher alcohols and acetate esters produced by the JET02-derived strains (strains that harbored *ATF1* genes from *S. kudriavzevii* or *S. uvarum*) showed significantly higher concentrations of 2-phenylethyl acetate in the phenylalanine-grown culture of the JET02Sk strain (**Figure 4A**). This strain, which harbored *SkATF1*, produced *c.* 2.7-fold higher concentrations than reference strain JET02Sc. With the other analyzed compounds only small and statistically insignificant differences were detected. In contrast, the JET03-derived strains (strains with introduced *SkATF2* or *SuATF2*) grown with the individual amino acids exhibited more differences during higher alcohols and acetate esters production. When grown with valine as the sole nitrogen source, the strain that carried *SkATF2* or *SuATF2* genes produced both corresponding derivatives, isobutanol and isobutyl acetate, in larger amounts than the strain with *ScATF2* (**Figure 4B**). Particularly, the isobutyl acetate concentrations were around threefold higher in both strains than that produced by JET03Sc. A similar upward trend in favor of JET03Sk and JET03Su was detected during isoamyl acetate production when their amino acidic precursor leucine was used as the nitrogen source. JET03Sk produced a twofold and JET03Su a 2.5-fold increase in the isoamyl acetate concentration. The phenylalanine-grown cultures of JET03Sk and JET03Su exhibited 2.2 and 1.9-fold larger amounts of 2-phenylethyl acetate than the reference JET03Sc.

### Comparison of the AATase Substrate Specificities

To determine and compare the AATase activities of Atf1p and Atf2p from the three different *Saccharomyces* species, the individual *ATF* genes were cloned into the plasmid pGREG526 and expressed in a host *S. cerevisiae* strain with the deleted *ATF1* and *ATF2* genes. In order to obtain sufficient AATase activity, the *ATF* genes were expressed under the control of the constitutive *TDH3* promoter. The host *S. cerevisiae* strain was also transformed with the empty pGREG526 vector as a negative control. In all assays with cell extracts of empty vector transformants, the concentrations of the measured acetate esters formed from the corresponding higher alcohols (isobutanol, isoamyl alcohol, and 2-phenylethanol) were below detection limit. This confirmed none or negligible alcohol acetyltransferase activity in these control strain cell extracts. The kinetic parameters (*K*_m_ and *V*_max_) were assessed for isoamyl alcohol as a substrate since isoamyl alcohol acetylation is known to be catalyzed only by Atf1p and Atf2p. To avoid the isoamyl acetate breakdown, the measured catalytic reaction product, *IAH1* gene that codifies ester hydrolase, was also deleted in the host strain (BY4741atf1atf2iah1). AATase activity was determined in cell extracts over a wide range of isoamyl alcohol concentrations (0.01–100 mM) and was measured by isoamyl acetate formation which was analyzed by head space gas chromatography. The activity measured in the cell extracts that expressed the *ATF1* orthologs showed typical Michaelis–Menten saturation kinetics (see Supplementary File 1). In contrast, the activity of all the AATases encoded by *ATF2* displayed an increasing tendency over the entire range of tested concentrations, without reaching saturation. Therefore, *K*_m_ and *V*_max_ were determined only for the enzymes encoded by the *ATF1* orthologs. The *K*_m_ and *V*_max_ values calculated for isoamyl alcohol displayed considerable differences among the individual Atf1 enzymes (**Table 4**). SkAtf1p showed almost twofold higher and SuAtf1p *c.* threefold higher *K*_m_ than ScAtf1p. Regarding *V*_max_, both SkAtf1p and SuAtf1p exhibited around twofold lower values than ScAtf1p.

**Table 4:** The kinetic parameters of SkAtf1p, SuAtf1p, and ScAtf1p measured in the cell extracts of the *S. cerevisiae* strain with *atf1 atf2 iah1* deletion.

Cell extract	*K*_m_ (mM)	*V*_max_ [nmol.min^-1^.(mg protein)^-1^]
CLpSkATF1	57.4 ± 7.1	4.80 ± 0.26
CLpSuATF1	92.9 ± 10.9	5.69 ± 0.35
CLpScATF1	32.2 ± 2.2	9.99 ± 0.24

**FIGURE 5:**
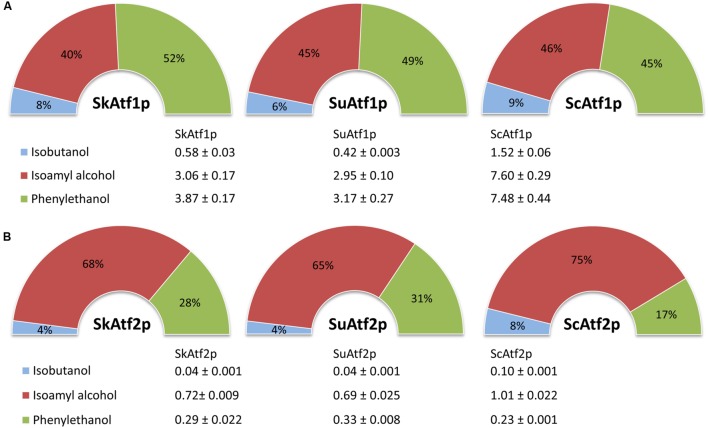
**The relative alcohol acetyltransferase activities of the Atf1 **(A)** and Atf2 **(B)** enzymes expressed as a percentage distributed among the total enzymatic activity of the measured substrates.** The presented values [nmol⋅min^-1^(mg protein)^-1^] are averages and mean deviations of three replicates. The activities of the different Atf1 and Atf2 enzymes were measured in the cell extracts of the *S. cerevisiae* strain with *atf1 atf2 iah1* deletion at a concentration of 60 mM for isobutanol, 100 mM for isoamyl alcohol and 30 mM for 2-phenylethanol.

To further characterize enzymatic properties, substrate specificities were compared for three different amino-acid-derived higher alcohols as follows: isobutanol, isoamyl alcohol, and 2-phenylethanol. Specific activities were analyzed at fixed substrate concentrations of 60 mM for isobutanol, 100 mM for isoamyl alcohol, and 30 mM for 2-phenylethanol. The cell extracts of all the strains that expressed the individual *ATF1* and *ATF2* orthologs exhibited activities for all the measured substrates. Interesting differences were observed between enzymes Atf1 and enzymes Atf2. When individual enzymatic activities were expressed as a percentage distributed among the total enzymatic activity of the measured substrates, enzymes Atf1 showed approximately equal percentage portions of the catalytic activities for isoamyl alcohol and 2-phenylethanol (**Figure 5A**). In contrast, enzymes Atf2 showed considerably stronger activity toward isoamyl alcohol than the other substrates. Moreover, while all three Atf1 enzymes displayed an almost identical percentage distribution of individual enzymatic activities, the proportion of the enzyme activity of SkAtf2p and SuAtf2p to 2-phenylethanol was almost twofold greater, and twofold lower to isobutanol than for ScAtf2p (**Figure 5B**).

## Discussion

Our previous study focused on a computational comparative analysis of the DNA sequences of the ortholog genes involved in higher alcohols and acetate ester formation in *S. cerevisiae*, *S. kudriavzevii*, and *S. uvarum* (Stribny et al., 2016). The individual changes noted in the *S. kudriavzevii* and *S. uvarum* sequences (with the *S. cerevisiae* orthologs taken as references) were evaluated by Grantham’s score, which quantifies the biochemical divergence between two amino acids on the basis of their molecular volume, polarity and composition (Grantham, 1974). Equally for *S. kudriavzevii* and *S. uvarum*, the alcohol acetyltransferases encoded by *ATF1* and *ATF2* clearly yielded the largest number of amino acid substitutions, as well as the highest Grantham scores (together with the 2-keto acid decarboxylase encoded by *ARO10*) among the 23 assessed sequences.

Hence, this work aimed to investigate and compare the effects of *ATF1* and *ATF2* from *S. kudriavzevii* and *S. uvarum* on the production of the aroma-active higher alcohols and acetate esters that derived from the corresponding amino acids. The gas chromatography analysis revealed increasing amounts of acetate esters for the strains that expressed genes *SkATF2* and *SuATF2*. One possible explanation is the amino acid substitutions observed during the aforementioned *in silico* analysis. Both sequences, SkAtf2p and SuAtf2p, revealed a large number of substitutions (20 and 25%, respectively) compared to ScAtf2p. Only four substitutions in SkAtf2p and five in SuAtf2p were classified as radical according to the Grantham scale. None of the substitutions were found in the two conserved regions, WRLICLP and HXXXD, hypothesized as parts of the active site (Nagasawa et al., 1998; D’Auria, 2006). Thus, the large number of total substitutions, or combinations of some, might play role in the differences observed during acetate ester production. While the Atf1p sequences also showed a large number of amino acid changes (SkAtf1p vs. ScAtf1p 17%, SuAtf1p vs. ScAtf1p 18%), the comparison of the strains that expressed the individual *ATF1* orthologs gave significant differences only for 2-phenylethyl acetate during the production of higher alcohols and acetate esters. Further research by mutagenesis assays could clarify a possible correlation between amino acid substitutions and the different higher alcohol and acetate ester productions.

The JET03-derived strains with introduced *SkATF2* and *SuATF2* (JET03Sk and JET03Su, respectively) also exhibited larger amounts of higher alcohols than JET03Sc. One possible explanation could be the catalytic activity of esterases that catalyzed ester breakdown, such as Iah1p (Fukuda et al., 2000; Lilly et al., 2006). In hypothetical terms, the superior concentrations of the higher alcohols produced by JET03Sk and JET03Su could result from Iah1p, which degraded the large amounts of acetate esters previously produced as a result of SkAtf2p and SuAtf2p activity. As previously described (Fukuda et al., 1998), the balance between ester-synthesizing and ester-degrading enzymes leads to an optimal balance between higher alcohols and acetate esters.

It has been shown that Atf1p and Atf2p in *S. cerevisiae* are able to transfer an activated acetate group to a wide variety of substrates with an alcohol group, and thus display wide substrate specificity for alcohol cosubstrates (Verstrepen et al., 2003b). Similarly, our findings showed wide substrate specificity for Atf1p and Atf2p from *S. kudriavzevii* and *S. uvarum*. The activities observed in the extracts of the cells that expressed the individual *ATF2* genes were an order of magnitude lower than the activities in the cell extracts that expressed *ATF1* genes. It has been hypothesized that Atf2p might be important for different metabolic processes other than Atf1p. For instance, Cauet et al. (1999) suggested that Atf2p plays a key role in the detoxification of 3β-hydroxysteroids. Our results also indicate that the Atf2 proteins from *S. kudriavzevii* and *S. uvarum* play only a secondary role in acetate ester formation compared to Atf1p, similarly to that observed in *S. cerevisiae* (Verstrepen et al., 2003b; Lilly et al., 2006).

Interesting differences were observed in the kinetic properties of the alcohol acetyltransferases among the Atf1p from *S. cerevisiae*, *S. kudriavzevii*, and *S. uvarum* toward isoamyl alcohol. Of the two cosubstrates, isoamyl alcohol was chosen for determining the kinetic properties because previous studies have shown alcohol to be a rate-limiting cosubstrate during alcohol acetyltransferases reactions (Minetoki et al., 1993). However, we did not observe any correlation between the different *K*_m_ values and isoamyl acetate formation. The strains that expressed the individual *ATF2* orthologs revealed marked differences in isoamyl acetate concentrations. However, unlike the kinetic data of the Atf1 enzymes, the individual Atf2p showed a linear increase of isoamyl acetate synthesis with isoamyl alcohol concentrations, and did not reach saturation, not even with the highest concentration (100 mM) used in the assay. Higher concentrations resulted in non-specific peaks, as detected by gas chromatography. As Malcorps et al. (1991) explained, one possible reason is that a high isoamyl alcohol concentration in the enzyme assay could modify the enzyme properties through conformational changes or denaturation, or could induce non-specific alcoholysis of the acetyl-enzyme intermediate. The higher isoamyl alcohol concentrations needed to determine the Atf2p kinetic properties indicated that the *K*_m_ values of the Atf2 enzymes were higher than those of Atf1p, which is in accordance with the observations made by Nagasawa et al. (1998). Regarding the Atf1 enzymes, the *K*_m_ (32.2 mM) for *S. cerevisiae* Atf1p calculated in our study was similar to that observed for *S. cerevisiae* sake strain Kyokai No. 7 (29.8 mM; Minetoki et al., 1993) and for *S. cerevisiae* beer strain NCYC 366 (25 mM; Malcorps and Dufour, 1992). These similar values indicate that the range of isoamyl alcohol concentrations used in our assays did not lead to the aforementioned modifications in the Atf1 enzymatic properties.

In a recent study we observed differences in the production of higher alcohols and acetate esters by *S. kudriavzevii* and *S. uvarum* compared to *S. cerevisiae* (Stribny et al., 2015). These differences could be explained by the different kinetic properties of the individual Atf1 enzymes and/or the activities of the Atf2 enzymes observed in the present work. Another reason could be variations in the expression levels of the *ATF* genes as the expression levels have been demonstrated to be an important factor for ester synthesis (Malcorps et al., 1991; Verstrepen et al., 2003b). For instance, Gamero et al. (2013) observed considerable differences in aroma production during wine fermentation by these three *Saccharomyces* species. The subsequent expression analysis revealed, besides others, the up-regulation of *ATF1* in *S. uvarum* and the up-regulation of *ATF2* in both *S. uvarum* and *S. kudriavzevii* compared to *S. cerevisiae* (Gamero et al., 2014). The existence of another as yet unknown enzyme with AATase activity in the yeast proteome has also been proposed (Malcorps and Dufour, 1992; Verstrepen et al., 2003b). Its putative effect on acetate ester production by the three different *Saccharomyces* species cannot be ruled out.

## Conclusion

The amino acid variations noted in the orthologous Atf1p and Atf2p of *S. kudriavzevii, S. uvarum*, and *S. cerevisiae* indicated a possible impact on the distinct properties of the enzymes characterized herein. Together with differences in gene expression levels, these distinct enzymatic properties appear to play an important role in the differences among these three *Saccharomyces* species during acetate ester formation. The knowledge on the important enzymes involved in aroma development by closely related *Saccharomyces* yeasts is of scientific as well as of applied interest, and uncover new possibilities to enhance biotechnological flavor production.

## Author Contributions

JS, RP-T, and AQ conceived and designed the experiments. JS performed the experiments. JS, AQ, and RP-T participated in the analysis and interpretation of the data. JS wrote the first manuscript version. AQ and RP-T participated in the final manuscript version. All authors read and approved the final manuscript version.

## Conflict of Interest Statement

The authors declare that the research was conducted in the absence of any commercial or financial relationships that could be construed as a potential conflict of interest.

## References

[B1] Beltran G. Novo M. Rozes N. Mas A. Guillamon J. M. (2004). Nitrogen catabolite repression in *Saccharomyces cerevisiae* during wine fermentations. *FEMS Yeast Res.* 4 625–632. 10.1016/j.femsyr.2003.12.004 15040951

[B2] Bolat I. Romagnoli G. Zhu F. B. Pronk J. T. Daran J. M. (2013). Functional analysis and transcriptional regulation of two orthologs of ARO10, encoding broad-substrate-specificity 2-oxo-acid decarboxylases, in the brewing yeast *Saccharomyces pastorianus* CBS1483. *FEMS Yeast Res.* 13 505–517. 10.1111/1567-1364.12051 23692465

[B3] Bradford M. M. (1976). A rapid and sensitive method for the quantitation of microgram quantities of protein utilizing the principle of protein-dye binding. *Anal. Biochem.* 72 248–254. 10.1016/0003-2697(76)90527-3 942051

[B4] Cauet G. Degryse E. Ledoux C. Spagnoli R. Achstetter T. (1999). Pregnenolone esterification in *Saccharomyces cerevisiae* – A potential detoxification mechanism. *Eur. J. Biochem.* 261 317–324. 10.1046/j.1432-1327.1999.00282.x 10103065

[B5] D’Auria J. C. (2006). Acyltransferases in plants: a good time to be BAHD. *Curr. Opin. Plant. Biol.* 9 331–340. 10.1016/j.pbi.2006.03.016 16616872

[B6] Dickinson J. R. Eshantha L. Salgado J. Hewlins M. J. E. (2003). The catabolism of amino acids to long chain and complex alcohols in *Saccharomyces cerevisiae*. *J. Biol. Chem.* 278 8028–8034. 10.1074/jbc.M211914200 12499363

[B7] Dickinson J. R. Harrison S. J. Dickinson J. A. Hewlins M. J. E. (2000). An investigation of the metabolism of isoleucine to active amyl alcohol in *Saccharomyces cerevisiae*. *J. Biol. Chem.* 275 10937–10942. 10.1074/jbc.275.15.10937 10753893

[B8] Dickinson J. R. Harrison S. J. Hewlins M. J. E. (1998). An investigation of the metabolism of valine to isobutyl alcohol in *Saccharomyces cerevisiae*. *J. Biol. Chem.* 273 25751–25756. 10.1074/jbc.273.40.25751 9748245

[B9] Dickinson J. R. Lanterman M. M. Danner D. J. Pearson B. M. Sanz P. Harrison S. J. (1997). A C-13 nuclear magnetic resonance investigation of the metabolism of leucine to isoamyl alcohol in *Saccharomyces cerevisiae*. *J. Biol. Chem.* 272 26871–26878. 10.1074/jbc.272.43.26871 9341119

[B10] Fujii T. Nagasawa N. Iwamatsu A. Bogaki T. Tamai W. Hamachi M. (1994). Molecular cloning, sequence analysis, and expression of the yeast alcohol acetyltransferase gene. *Appl. Environ. Microbiol.* 60 2786–2792. 8085822 10.1128/aem.60.8.2786-2792.1994 PMC201724

[B11] Fukuda K. Kiyokawa Y. Yanagiuchi T. Wakai Y. Kitamoto K. Inoue Y. (2000). Purification and characterization of isoamyl acetate-hydrolyzing esterase encoded by the IAH1 gene of *Saccharomyces cerevisiae* from a recombinant *Escherichia coli*. *Appl. Microbiol. Biotechnol.* 53 596–600. 10.1007/s002530051662 10855721

[B12] Fukuda K. Yamamoto N. Kiyokawa Y. Yanagiuchi T. Wakai Y. Kitamoto K. (1998). Balance of activities of alcohol acetyltransferase and esterase in *Saccharomyces cerevisiae* is important for production of isoamyl acetate. *Appl. Environ. Microbiol.* 64 4076–4078. 9758847 10.1128/aem.64.10.4076-4078.1998 PMC106606

[B13] Gamero A. Belloch C. Ibanez C. Querol A. (2014). Molecular analysis of the genes involved in aroma synthesis in the species *S. cerevisiae*, *S. kudriavzevii* and *S. bayanus* var. uvarum in winemaking conditions. *Plos ONE* 9:e97626 10.1371/journal.pone.0097626 PMC4031168 24854353

[B14] Gamero A. Tronchoni J. Querol A. Belloch C. (2013). Production of aroma compounds by cryotolerant *Saccharomyces* species and hybrids at low and moderate fermentation temperatures. *J. Appl. Microbiol.* 114 1405–1414. 10.1111/jam.12126 23294204

[B15] Gietz R. D. Woods R. A. (2002). Transformation of yeast by lithium acetate/single-stranded carrier DNA/polyethylene glycol method. *Methods Enzymol.* 350 87–96. 10.1016/S0076-6879(02)50957-5 12073338

[B16] Goldstein A. L. McCusker J. H. (1999). Three new dominant drug resistance cassettes for gene disruption in *Saccharomyces cerevisiae*. *Yeast* 15 1541–1553. 10.1002/(sici)1097-0061(199910)15:14<1541::aidyea476>3.0.co;2-k 10514571

[B17] Grantham R. (1974). Amino acid difference formula to help explain protein evolution. *Science* 185 862–864. 10.1126/science.185.4154.862 4843792

[B18] Hazelwood L. A. Daran J. M. van Maris A. J. A. Pronk J. T. Dickinson J. R. (2008). The Ehrlich pathway for fusel alcohol production: a century of research on *Saccharomyces cerevisiae* metabolism. *Appl. Environ. Microbiol.* 74 2259–2266. 10.1128/aem.02625-2627 18281432 PMC2293160

[B19] Jansen G. Wu C. L. Schade B. Thomas D. Y. Whiteway M. (2005). Drag&Drop cloning in yeast. *Gene* 344 43–51. 10.1016/j.gene.2004.10.016 15656971

[B20] Kaneko Y. Banno I. (1991). Re-examination of *Saccharomyces bayanus* strains by DNA-DNA hybridization and electrophoretic karyotyping. *IFO Res. Comm.* 15 30–41.

[B21] Lambrechts M. G. Pretorius I. S. (2000). Yeast and its importance to wine aroma – a review. *S. Afr. J. Enol. Viticul.* 21 97–125.

[B22] Lilly M. Bauer F. F. Lambrechts M. G. Swiegers J. H. Cozzolino D. Pretorius I. S. (2006). The effect of increased yeast alcohol acetyltransferase and esterase activity on the flavour profiles of wine and distillates. *Yeast* 23 641–659. 10.1002/yea.1382 16845703

[B23] Malcorps P. Cheval J. M. Jamil S. Dufour J. P. (1991). A new model for the regulation of ester synthesis by alcohol acetyltransferase in *Saccharomyces cerevisiae* during fermentation. *J Am. Soc. Brew. Chem.* 49 47–53.

[B24] Malcorps P. Dufour J. P. (1992). Short-chain and medium -chain aliphatic-ester synthesis in *Saccharomyces cerevisiae*. *Eur. J. Biochem.* 210 1015–1022. 10.1111/j.1432-1033.1992.tb17507.x 1483449

[B25] Minetoki T. Bogaki T. Iwamatsu A. Fujii T. Hamachi M. (1993). The purification, properties and internal peptide sequences of alcohol acetytransferase isolated from *Saccharomyces cerevisiae* Kyokai No. 7. *Biosci. Biotechnol. Biochem.* 57 2094–2098. 10.1271/bbb.57.2094 7764365

[B26] Nagasawa N. Bogaki T. Iwamatsu A. Hamachi M. Kumagai C. (1998). Cloning and nucleotide sequence of the alcohol acetyltransferase II gene (ATF2) from *Saccharomyces cerevisiae* Kyokai No. 7. *Biosci. Biotechnol. Biochem.* 62 1852–1857. 10.1271/bbb.62.1852 9836419

[B27] Nykanen L. (1986). Formation and occurence of flavor compounds in wine and distilled alcoholic beverages. *Am. J. Enol. Vitic.* 37 84–96.

[B28] Querol A. Huerta T. Barrio E. Ramon D. (1992). Dry yeast-strain for use in fermentation of Alicante wines – selection and DNA patterns. *J. Food Sci.* 57 183–185. 10.1111/j.1365-2621.1992.tb05451.x

[B29] Riou C. Nicaud J. M. Barre P. Gaillardin C. (1997). Stationary-phase gene expression in *Saccharomyces cerevisiae* during wine fermentation. *Yeast* 13 903–915. 10.1002/(sici)1097-0061(199708)13:10<903::aid-yea145>3.3.co;2-t 9271106

[B30] Rojas V. (2002). *Actividades Esterásicas en Levaduras Vínicas.* Ph.D., thesis, University of Valencia Spain.

[B31] Rojas V. Gil J. V. Manzanares P. Gavara R. Pinaga F. Flors A. (2002). Measurement of alcohol acetyltransferase and ester hydrolase activities in yeast extracts. *Enzyme Microb. Technol.* 30 224–230. 10.1016/s0141-0229(01)00483-485

[B32] Stribny J. Gamero A. Perez-Torrado R. Querol A. (2015). *Saccharomyces kudriavzevii* and *Saccharomyces uvarum* differ from *Saccharomyces cerevisiae* during the production of aroma-active higher alcohols and acetate esters using their amino acidic precursors. *Int. J. Food Microbiol.* 205 41–46. 10.1016/j.ijfoodmicro.2015.04.003 25886016

[B33] Stribny J. Romagnoli G. Perez-Torrado R. Daran J. M. Querol A. (2016). Characterisation of the broad substrate specificity 2-keto acid decarboxylase Aro10p of *Saccharomyces kudriavzevii* and its implication in aroma development. *Microb. Cell Fact.* 15 51 10.1186/s12934-016-0449-z PMC4789280 26971319

[B34] Styger G. Prior B. Bauer F. F. (2011). Wine flavor and aroma. *J. Ind. Microbiol. Biotechnol.* 38 1145–1159. 10.1007/s10295-011-1018-1014 21786136

[B35] Uber-Garcia G. (2005). *Modificación Genética De Levaduras Vínicas Industriales Par Mejorar La Producción De Aroma Secundario.* Ph.D., thesis, University of Valencia Spain.

[B36] Verstrepen K. J. Derdelinckx G. Dufour J. P. Winderickx J. Thevelein J. M. Pretorius I. S. (2003a). Flavor-active esters: Adding fruitiness to beer. *J. Biosci. Bioeng.* 96 110–118. 10.1016/s1389-1723(03)90112-90115 16233495

[B37] Verstrepen K. J. Van Laere S. D. M. Vanderhaegen B. M. P. Derdelinckx G. Dufour J. P. Pretorius I. S. (2003b). Expression levels of the yeast alcohol acetyltransferase genes ATF1, Lg-ATF1, and ATF2 control the formation of a broad range of volatile esters. *Appl. Environ. Microbiol.* 69 5228–5237. 10.1128/aem.69.9.5228-5237.2003 12957907 PMC194970

[B38] Welsh F. W. Murray W. D. Williams R. E. (1989). Microbiological and enzymatic production of flavor and fragrance chemicals. *Crit. Rev. Biotechnol.* 9 105–169. 10.3109/07388558909040617

[B39] Yoshioka K. Hashimoto N. (1981). Ester formation by alcohol acetyltransferase from brewers’ yeast. *Agri. Biol. Chem.* 45 2183–2190.

