# Supplementary material for: Differences in Enzymatic Properties of the Saccharomyces kudriavzevii and Saccharomyces uvarum Alcohol Acetyltransferases and Their Impact on Aroma-Active Compounds Production
Source: Front Microbiol. 2016 Jun 7;7:897. doi: 10.3389/fmicb.2016.00897 (PMC4894917; doi:10.3389/fmicb.2016.00897)
Supplement: Supplementary file 1 [file Data_Sheet_1.DOCX]

**The data to calculate Km and Vmax were fitted with GraphPad Prism 4.0 software by using a nonlinear regression of the Michaelis-Menten equation.**

**Here are the results in more detail:**

| **Michaelis-Menten** |  |  |  |
| --- | --- | --- | --- |
| **Best-fit values** |  |  |  |
| Vmax | 0.4429 | 0.2128 | 0.252 |
| Km | 32.17 | 57.42 | 92.89 |
| **Std. Error** |  |  |  |
| Vmax | 0.01097 | 0.01171 | 0.01562 |
| Km | 2.173 | 7.119 | 10.86 |
| **95% Confidence Intervals** | |  |  |
| Vmax | 0,4202 to 0,4657 | 0,1885 to 0,2371 | 0,2196 to 0,2844 |
| Km | 27,66 to 36,67 | 42,66 to 72,19 | 70,38 to 115,4 |
| **Goodness of Fit** |  |  |  |
| Degrees of Freedom | 22 | 22 | 22 |
| R square | 0.9961 | 0.991 | 0.9952 |
| Absolute Sum of Squares | 0.001164 | 0.0004161 | 0.0002026 |
| Sy.x | 0.007273 | 0.004349 | 0.003035 |
